# Association between the relative abundance of gastric microbiota and the risk of gastric cancer: a case-control study

**DOI:** 10.1038/s41598-019-50054-x

**Published:** 2019-09-19

**Authors:** Madhawa Neranjan Gunathilake, Jeonghee Lee, Il Ju Choi, Young-Il Kim, Yongju Ahn, Chanhyeok Park, Jeongseon Kim

**Affiliations:** 1 Department of Cancer Control and Population Health, Graduate School of Cancer Science and Policy, Goyang-si, 10408 Gyeonggi-do South Korea; 2 Department of Cancer Biomedical Science, Graduate School of Cancer Science and Policy, Goyang-si, 10408 Gyeonggi-do South Korea; 3 Center for Gastric Cancer, National Cancer Center Hospital, National Cancer Center, Goyang-si, 10408 Gyeonggi-do South Korea; 4 Microbiome Division, Theragen Etex, 145 Gwanggyo-ro, Gyeongtong-gu, Suwon-si, Gyeonggi-do 16229 South Korea

**Keywords:** Cancer epidemiology, Gastric cancer

## Abstract

The human gut hosts a diverse community of bacteria referred to as the gut microbiome. We investigated the association between the relative abundance of gastric microbiota and gastric cancer (GC) risk in a Korean population. The study participants included 268 GC patients and 288 controls. DNA was extracted from gastric biopsies, and 16S rRNA gene analysis was performed. Unconditional logistic regression models were used to observe the associations. Of the participants, those who had the highest level (highest tertile) of relative *Helicobacter pylori* and *Propionibacterium acnes* abundances showed a significantly higher risk for GC after adjusting for potential confounding variables (odds ratio (OR) = 1.86, 95% confidence interval (CI) = 1.17–2.97, p for trend = 0.017 and OR = 4.77, 95% CI = 2.94–7.74, p for trend <0.001, respectively). Subjects who carried *Prevotella copri* had a significantly higher risk of GC than noncarriers (OR = 2.54, 95% CI = 1.42–4.55, p for trend = 0.002). There was a lower risk of GC in subjects carrying *Lactococcus lactis* than in noncarriers (OR = 0.21, 95% CI = 0.10–0.44, p for trend <0.001). *H. pylori, P. acnes* and *P. copri* are strong risk factors, whereas *L. lactis* is a protective factor, for GC development in Koreans. Further microbiome studies are warranted to verify the findings of the current study.

## Introduction

Gastric cancer (GC) ranks as the fifth leading cancer type, and it has been identified as one of the main causes of cancer-related deaths in the world^1^. The incidence of GC in eastern Asia, including Korea, is the highest worldwide, which is over 4 times higher than the rates in Western Europe^2^. It has been reported that the age-adjusted incidence rate of GC was 33.8 per 100,000 in Korea^3^. According to a prediction of cancer incidence and mortality in Korea, GC accounts for a remarkable proportion of the overall cancer burden because it is the second most common type of cancer among Koreans^4^. Recent studies about the human microbiome demonstrate a surge in interest in the context of disease, particularly in gastrointestinal cancers^5^. It is a known fact that there are various types of bacteria in different body sites, which are colloquially referred to as normal flora. This microbiota has the potential to maintain human health by interacting with the human body and can be considered pathological for the development of certain diseases^5^.

Due to the complex and dynamic nature of the human gastrointestinal microbiota, it is recently considered as a metabolically active organ and the complex nature of it evidently regulates gastrointestinal homeostasis by interacting with immune cells^6^. The normal flora in the gastrointestinal tract supports several processes, including the host mucosal immune response, energy metabolism, pathogen elimination, and cancer development^7^. It is widely implicated that human gut bacteria play a crucial role in the etiology of gastrointestinal cancers, particularly GC due to dysbiosis^8^. Dysbiosis is a condition in which there is an imbalance in the gastrointestinal microbiota, which consequently leads to several pathological conditions, specifically GC. Furthermore, gastric microbial community profiling revealed that dysbiosis of gut microbiota is associated with GC or precancerous lesions^9^.

The relationship between particular microbial pathogens and carcinogenesis has been the subject of exploration in the context of systems epidemiology. A considerable number of studies have focused on individual pathogens, such as *Helicobacter pylori (H. pylori)*, and their ability to initiate disease conditions, such as gastritis or GC^10^. Of the numerous risk factors associated with GC occurrence, *H. pylori* infection plays a pivotal role^11^. It is an established fact that *H. pylori* infection is widespread in East Asia, which is associated with GC development^12^. Thus, it has been extensively suggested that reduction in chronic *H. pylori* infection is a useful strategy for preventing GC. Notably, the identification of specific microbial species that are associated with various disease conditions, particularly cancers pertaining to the gastrointestinal tract, has been achieved with the expansion of advance sequencing technologies. It has been suggested that colonization of non-*H. pylori* bacteria in the stomach can also stimulate GC risk^13^. Thus, understanding how dysbiosis influences host metabolic reactions and inflammatory responses is critical to defining the roles specific components of the microbiota play in carcinogenesis.

However, there is a paucity of data regarding the association between the gastric microbiota and GC. As it is well documented that the composition of the microbiota shapes immune responses at local and systemic levels, as well as inflammatory signaling related to GC development, it is necessary to investigate the gastric microbiota-associated alterations that may influence GC development. Moreover, it is necessary to explore the evidence related to this association based on epidemiological studies because various experimental approaches have already been employed to elucidate the microbiota profiles of GC patients^14^. Therefore, in the present case-control study, we aimed to investigate the association between the relative abundance of the gastric microbiota components and the risk of GC in a Korean population.

## Results

### General characteristics of the study population

Table 1 presents the general characteristics of the study participants with and without GC. The proportion of current smokers was higher in the patient group (29.1%) than in the control group (17.7%), and the patients were more likely to have a family history of GC (p = 0.003). The patients engaged in exercise less regularly (p < 0.001), were less educated (p < 0.001), and exhibited lower employment rates (p = 0.037) and lower monthly incomes (p < 0.001) than the controls. The proportion of *H. pylori* infection among the patients (99.6%) was higher than among the controls (93.4%). The patients had a higher energy intake than the controls (p < 0.001). The Shannon index, which represents the alpha diversity, was significantly higher in the controls than in the patients (p < 0.001).

**Table 1:** General characteristics of the study population.

Variable	All (n = 556)			Male (n = 353)			Female (n = 203)		
	Control (n = 288)	Cases (n = 268)	p-value	Control (n = 181)	Cases (n = 172)	p-value	Control (n = 107)	Cases (n = 96)	p-value
Age (y)	51.53 ± 7.21	53.68 ± 9.60	**0.003**	52.07 ± 6.46	54.69 ± 8.86	**0.002**	50.62 ± 8.29	51.86 ± 10.59	0.355
<50	114 (39.58)	93 (34.70)	0.234	65 (35.91)	52 (30.23)	0.257	49 (45.79)	41 (42.71)	0.657
≥50	174 (60.42)	175 (65.30)		116 (64.09)	120 (69.77)		58 (54.21)	55 (57.29)	
Gender [n (%)]			0.745						
Male	181 (62.85)	172 (64.18)							
Female	107 (37.15)	96 (35.82)							
Body Mass index (kg/m²) [n (%)]	23.99 ± 3.11	23.91 ± 3.02	0.747	24.48 ± 3.04	24.30 ± 2.85	0.573	23.18 ± 3.07	23.21 ± 3.20	0.939
<23	113 (39.24)	107 (39.93)	0.863	58 (32.04)	58 (33.72)	0.931	55 (51.40)	49 (51.04)	0.802
23–25	81 (28.13)	70 (26.12)		51 (28.18)	46 (26.74)		30 (28.04)	24 (25.00)	
≥25	94 (32.64)	91 (33.96)		72 (39.78)	68 (39.53)		22 (20.56)	23 (23.96)	
Missing									
Smoking status [n (%)]			**0.006**			**0.006**			**0.243**
Current smoker	51 (17.71)	78 (29.10)		50 (27.62)	75 (43.60)		1 (0.93)	3 (3.13)	
Ex-smoker	98 (34.03)	80 (29.85)		95 (52.49)	74 (43.02)		3 (2.80)	6 (6.25)	
Nonsmoker	139 (48.26)	109 (40.67)		36 (19.89)	23 (13.37)		103 (96.26)	86 (89.58)	
Missing	0 (0.00)	1 (0.37)					0 (0.00)	1 (1.04)	
Alcohol consumption [n (%)]			0.559			0.618			0.860
Current drinker	184 (63.89)	163 (60.82)		137 (75.69)	123 (71.51)		47 (43.93)	40 (41.67)	
Ex-drinker	21 (7.29)	26 (9.70)		17 (9.39)	21 (12.21)		4 (3.74)	5 (5.21)	
Nondrinker	83 (28.82)	78 (29.10)		27 (14.92)	28 (16.28)		56 (52.34)	50 (52.08)	
Missing	0 (0.00)	1 (0.37)					0 (0.00)	1 (1.04)	
Family history of gastric cancer			**0.003**			**0.015**			0.112
Yes	34 (11.81)	56 (20.90)		25 (13.81)	41 (23.84)		9 (8.41)	15 (15.63)	
No	254 (88.19)	211 (78.73)		156 (86.19)	130 (75.58)		98 (91.59)	81 (84.38)	
Missing	0 (0.0)	1 (0.37)							
Regular exercise [n (%)]			**<0.001**			0.079			**<0.001**
Yes	150 (52.08)	95 (35.45)		89 (49.17)	69 (40.12)		61 (57.01)	26 (27.08)	
No	137 (47.57)	173 (64.55)		91 (50.28)	103 (59.88)		46 (42.99)	70 (72.92)	
Missing	1 (0.35)	0 (0.00)		1 (0.55)	0 (0.00)				
Educational level [n (%)]			**<0.001**			**<0.001**			**0.001**
Middle school	42 (14.58)	92 (34.33)		25 (13.81)	58 (33.72)		17 (15.89)	34 (35.42)	
High school	86 (29.86)	116 (43.28)		43 (23.76)	77 (44.77)		43 (40.19)	39 (40.63)	
College or more	148 (51.39)	58 (21.64)		103 (56.91)	36 (20.93)		45 (42.06)	22 (22.92)	
Missing	12 (4.17)	2 (0.75)		10 (5.52)	1 (0.58)		2 (1.87)	1 (1.04)	
Occupation [n (%)]			**0.037**			**0.004**			**0.017**
Group1:Professionals, administrative management	60 (20.83)	44 (16.42)		45 (24.86)	37 (21.51)		15 (14.02)	7 (7.29)	
Group2:Office, Sales and service positions	98 (34.03)	72 (26.87)		74 (40.88)	46 (26.74)		24 (22.43)	26 (27.08)	
Group3:Agriculture, laborer	47 (16.32)	65 (24.25)		43 (23.76)	51 (29.65)		4 (3.74)	14 (14.58)	
Group4:Unemployment and others	83 (28.82)	85 (31.72)		19 (10.50)	37 (21.51)		64 (59.81)	48 (50.00)	
Missing	0 (0.00)	2 (0.75)		0 (0.00)	1 (0.58)		0 (0.00)	1 (1.04)	
Marital status [n (%)]			0.319			0.249			0.864
Married	245 (85.07)	234 (87.31)		157 (86.74)	155 (90.12)		88 (82.24)	79 (82.29)	
Others (single, divorced, separated, widowed, cohabitating)	43 (14.93)	32 (11.94)		24 (13.26)	16 (9.30)		19 (17.76)	16 (16.67)	
Missing	0 (0.00)	2 (0.75)		0 (0.00)	1 (0.58)		0 (0.00)	1 (1.04)	
Monthly income[n (%)]*			**<0.001**			**<0.001**			0.084
<200	46 (15.97)	79 (29.48)		21 (11.60)	49 (28.49)		25 (23.36)	30 (31.25)	
200–400	114 (39.58)	101 (37.69)		80 (44.20)	70 (40.70)		34 (31.78)	31 (32.29)	
≥400	110 (38.19)	59 (22.01)		64 (35.36)	34 (19.77)		46 (42.99)	25 (26.04)	
Missing	18 (6.25)	29 (10.82)		16 (8.84)	19 (11.05)		2 (1.87)	10 (10.42)	
*H. pylori* infection			**<0.001**			**0.008**			**0.004**
Positive	269 (93.40)	267 (99.63)		171 (94.48)	171 (99.42)		98 (91.59)	96 (100.00)	
Negative	19 (6.60)	1 (0.37)		10 (5.52)	1 (0.58)		9 (8.41)	0 (0.00)	
Missing									
Supplements use [n(%)]			0.416			0.342			0.892
Yes	159 (55.21)	157 (58.58)		92 (50.83)	96 (55.81)		67 (62.62)	61 (63.54)	
No	127 (44.10)	109 (40.67)		87 (48.07)	74 (43.02)		40 (37.38)	35 (36.46)	
Missing	2 (0.69)	2 (0.75)		2 (1.10)	2 (1.16)				
Lauren’s classification			NA			NA			NA
Intestinal	NA	105 (39.18)		NA	89 (51.74)		NA	16 (16.67)	
Diffuse	NA	109 (40.67)		NA	51 (29.65)		NA	58 (60.42)	
Mixed	NA	36 (13.43)		NA	21 (12.21)		NA	15 (15.63)	
Missing	NA	18 (6.72)		NA	11 (6.40)		NA	7 (7.29)	
Total Energy intake (Kcal/day)	1766.35 ± 554.67	1934.24 ± 624.91	**<0.001**	1839.30 ± 542.53	2057.70 ± 643.65	**0.001**	1642.95 ± 555.62	1713.03 ± 524.18	0.358
Shannon index	1.38 ± 1.09	1.06 ± 0.82	**<0.001**	1.37 ± 1.03	1.16 ± 0.86	**0.013**	1.40 ± 1.17	0.89 ± 0.72	**<0.001**

Values are expressed as mean ± standard deviation (SD) or n (%). *Unit is 10,000 Won in Korean currency. Exchange rate 1 US$ = 1122 Korean Won (February 2019). **p values were calculated by using Student’s t-test for continuous variables and chi-square test for categorical variables.

### Comparison of relative abundance and diversity indices

Table 2 presents the mean relative abundance comparison results between the patients with GC and controls. We found that patients with GC had a higher relative abundance of *Helicobacteraceae, Propionibacteriaceae*, and *Prevotellaceae* than the healthy subjects at the family level (p adjusted for false discovery rate (FDR) = 0.025, <0.001, 0.013, respectively). At the genus level, the relative abundances of *Helicobacter, Propionibacterium*, and *Prevotella* were higher in the patients than in the controls (p adjusted for FDR = 0.025, <0.001, 0.013, respectively) whereas the relative abundance of *Lactococcus* was higher in the controls than in the patients (p adjusted for FDR < 0.001). Regarding the species level, we found that the patients had higher relative abundances of *H. pylori, Propionibacterium acnes* (*P. acnes*), and *Prevotella copri* (*P. copri*) than the controls (p adjusted for FDR = 0.035, 0.002, and 0.049, respectively), while the relative abundance of *Lactococcus lactis* (*L. lactis*) was higher in the healthy controls than in the patients (p adjusted for FDR = 0.003).

**Table 2:** Mean relative abundance of selected family, genus, and species between the GC cases and controls.

Taxonomy level	Control (288)		Case (268)		p-value^a^
	Mean	SD	Mean	SD	
Taxonomy Family					
*Helicobacteraceae*	0.809	0.26	0.856	0.188	0.025
*Propionibacteriaceae*	0.000039	0.00008	0.0000932	0.000192	<0.001
*Prevotellaceae*	0.0309	0.0764	0.0162	0.0397	0.013
Taxonomy Genus					
*Helicobacter*	0.809	0.26	0.856	0.188	0.025
*Propionibacterium*	0.000035	0.000076	0.000091	0.00019	<0.001
*Prevotella*	0.024	0.062	0.012	0.031	0.013
*Lactococcus*	4.96E-06	0.000017	9.07E-07	4.91E-06	<0.001
Taxonomy Species					
*Helicobacter pylori*	0.809	0.26	0.857	0.188	0.035
*Propionibacterium acnes*	0.000035	0.000075	0.000091	0.00019	0.002
*Prevotella copri*	2.57E-06	0.000011	4.45E-06	0.000012	0.049
*Lactococcus lactis*	4.90E-06	0.000017	9.07E-07	4.91E-06	0.003

^a^p values corrected by false discovery rate (FDR).

**Figure 1:**
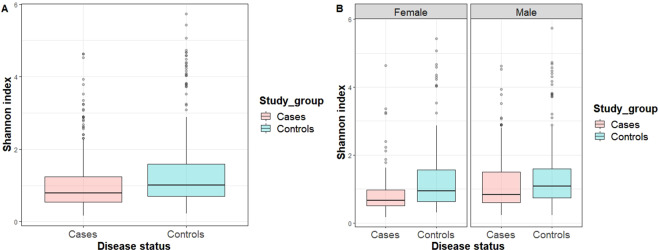
Box plot of the Shannon index. (**A**) Box plot of the Shannon index for the cases and controls (p < 0.001). (**B**) Box plot of the Shannon index for cases and controls by sex (p = 0.013 for male and p < 0.001 for female).

**Figure 2:**
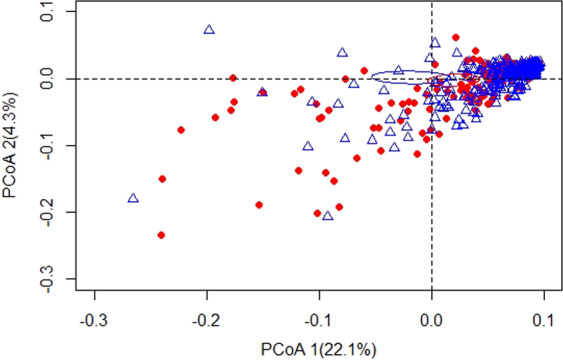
Principal coordinate analysis (PCoA) plot of the Bray-Curtis distance. The blue triangles indicate the controls while the red dots indicate the cases. The blue and red ellipses represent where 95% of data belong to the controls and cases, respectively, at 95% significance.

Figure 1A presents a box plot for the Shannon index for the entire study population. We observed that the Shannon index was significantly higher in the controls than in the patients (p < 0.001) in the entire study population. Figure 1B shows the box plots for the Shannon index by sex. We found that the Shannon index was significantly higher in controls than in male or female patients (p = 0.013 and p < 0.001, respectively). Figure 2 represents a principal coordinates analyses (PCoA) plot of the Bray-Curtis distance based on the operational taxonomic units (OTU) relative abundance table at the species level. The blue triangles represent the controls, while the red dots represent the patient. The blue and red ellipses represent cases in which 95% of the data belong to the controls and patients at a 95% significance level. The 2-D plot of the first two principal coordinates shows a marked divergence between the GC patients and the healthy controls. The total diversity captured by the first two principal coordinates was 26.4%. The microbiota composition of the patients with GC was significantly different from that of the healthy controls (analysis of similarity (ANOSIM) R = 0.006, p = 0.030) according to the Bray-Curtis dissimilarity measure.

### Association of candidate species with GC risk and evaluation of their abundance for GC diagnosis

Table 3 shows the association between the relative abundance of candidate bacterial species and GC risk. The subjects, who had a higher relative abundance of *H. pylori* showed a significantly higher risk of GC in model II than the subjects who had a lower relative abundance (odd ratio (OR) = 1.86, 95% confidence interval (CI) = 1.17–2.97, p-trend = 0.017). The females who had a higher relative abundance of *H. pylori* showed a significantly higher risk of GC than the females who had a lower relative abundance (OR = 3.36, 95% CI = 1.41–8.00, p-trend = 0.008). A positive association between the relative abundance of *H. pylori* and GC was observed in males, although the results were not significant.

**Table 3:** Associations between the relative bacterial species abundances and GC risk.

Species	No. of controls	No. of cases	Model I OR (95% CI)	Model II OR (95% CI)
*Helicobacter pylori*				
All				
<0.861	95 (33.0)	73 (27.2)	1.00	1.00
0.861–0.931	96 (33.3)	77 (28.7)	1.04 (0.68–1.60)	1.22 (0.75–1.99)
> = 0.931	97 (33.7)	118 (44.0)	1.58 (1.05–2.38)	1.86 (1.17–2.97)
p for trend			0.058	0.017
Male				
<0.862	61 (33.7)	58 (33.7)	1.00	1.00
0.862–0.930	60 (33.2)	48 (27.9)	0.84 (0.50–1.42)	1.06 (0.57–1.98)
> = 0.930	60 (33.2)	66 (38.4)	1.16 (0.70–1.91)	1.59 (0.87–2.91)
p for trend			0.761	0.195
Female				
<0.858	35 (32.7)	14 (14.6)	1.00	1.00
0.858–0.936	36 (33.6)	34 (35.4)	2.36 (1.09–5.14)	2.15 (0.88–5.27)
> = 0.936	36 (33.6)	48 (50.0)	3.33 (1.57–7.10)	3.36 (1.41–8.00)
p for trend			0.002	0.008
*Propionibacterium acnes*				
All				
<0.000006	117 (40.6)	47 (17.5)	1.00	1.00
0.000006–0.000029	82 (28.5)	51 (19.0)	1.55 (0.95–2.52)	1.58 (0.92–2.71)
> = 0.000029	89 (30.9)	170 (63.4)	4.76 (3.11–7.27)	4.48 (2.79–7.21)
p for trend			<0.001	<0.001
Male				
<0.00001	63 (34.8)	32 (18.6)	1.00	1.00
0.00001–0.000031	57 (31.5)	33 (19.2)	1.14 (0.62–2.09)	1.29 (0.64–2.59)
> = 0.000031	61 (33.7)	107 (62.2)	3.45 (2.03–5.86)	3.03 (1.62–5.66)
p for trend			<0.001	<0.001
Female				
0	43 (40.2)	12 (12.5)	1.00	1.00
0–0.000022	27 (25.2)	16 (16.7)	2.12 (0.87–5.17)	2.89 (1.03–8.11)
> = 0.000022	37 (34.6)	68 (70.8)	6.59 (3.10–14.00)	8.19 (3.41–19.65)
p for trend			<0.001	<0.001
*Prevotella copri*				
All				
0 (noncarriers)^a^	263 (91.3)	214 (79.9)	1.00	1.00
>0 (carriers)^a^	25 (8.7)	54 (20.2)	2.65 (1.60–4.41)	2.39 (1.36–4.19)
Male				
0 (noncarriers)^a^	165 (91.2)	133 (77.3)	1.00	1.00
>0 (carriers)^a^	16 (8.8)	39 (22.7)	3.02 (1.62–5.65)	2.78 (1.32–5.85)
Female				
0 (noncarriers)^a^	98 (91.6)	81 (84.4)	1.00	1.00
>0 (carriers)^a^	9 (8.4)	15 (15.6)	2.02 (0.84–4.85)	1.80 (0.66–4.92)
*Lactococcus lactis*				
All				
0 (noncarriers)^a^	238 (82.6)	257 (95.9)	1.00	1.00
>0 (carriers)^a^	50 (17.4)	11 (4.1)	0.20 (0.10–0.40)	0.19 (0.10–0.39)
Male				
0 (noncarriers)^a^	151 (83.4)	165 (95.9)	1.00	1.00
>0 (carriers)^a^	30 (16.6)	7 (4.1)	0.21 (0.10–050)	0.22 (0.08–0.57)
Female				
0 (noncarriers)^a^	87 (81.3)	92 (95.8)	1.00	1.00
>0 (carriers)^a^	20 (18.7)	4 (4.2)	0.19 (0.06–0.58)	0.18 (0.05–0.60)

Model I: Crude.

Model II: Adjusted for age, family history of GC, regular exercise, education, occupation, income, total energy intake.

^a^If more than one-third of the subjects had a relative abundance of zero, that bacterial species was categorized into two groups (noncarriers and carriers) based on the median distribution of the controls.

Those who had a higher relative abundance of *P. acnes* showed a significantly higher risk for GC than those in the lowest relative abundance group among the entire study population (OR = 4.48, 95% CI = 2.79–7.21, p-trend ≤ 0.001), in males (OR = 3.03, 95% CI = 1.62–5.66, p-trend ≤ 0.001) and females (OR = 8.19, 95% CI = 3.41–19.65, p-trend ≤ 0.001). Regarding *P. copri*, those who carried this species showed a significantly higher risk of GC than the noncarriers among the entire population (OR = 2.39, 95% CI = 1.36–4.19) and in males (OR = 2.78, 95% CI = 1.32–5.85). The subjects who carried *L. lactis* showed a significantly lower risk of GC than the noncarriers among the entire population (OR = 0.19, 95% CI = 0.10–0.39), in males (OR = 0.22, 95% CI = 0.08–0.57) and in females (OR = 0.18, 95% CI = 0.05–0.60) (Table 3).

Based on principal component analysis (PCA), two linear combinations of four species were obtained using Eigen value greater than 1.00. *H. pylori* and *P. acnes* were identified as dominant bacterial species in components 1 and 2, respectively, based on the principal component loadings. Approximately 63.0% of the variation was explained by the first two principal components. According to the linear discriminant analysis, the coefficients for the linear discriminants for PC1 (*H. pylori* dominant) and PC2 (*P. acnes* dominant) were −0.07 and 0.97, respectively. We investigated whether the combination of these four bacterial species could demonstrate better predictive ability using a receiver operating characteristic (ROC) curve and area under curve (AUC) analyses. An analysis using two linear combinations of four bacterial species showed 79.7% sensitivity and 67.1% specificity. The AUC was 77.7% indicating that there is a 77.7% chance that the model will be able to distinguish between positive and negative classes (Supplementary Fig. S1). The best cutoff point was chosen as 0.492 in order to find a balance between sensitivity and specificity.

## Discussion

In this case-control study involving 556 participants (268 patients and 288 controls), we observed that the relative abundances of the *H. pylori*, *P. acnes* and *P. copri* species were significantly higher in the patients than in the controls, whereas the relative abundance of *L. lactis* was significantly higher in the controls than in the patients. Generally, the subjects who had a high relative abundance of the *H. pylori*, *P. acnes* and *P. copri* species showed a significantly higher risk of GC. In contrast, those who had a high relative abundance of *L. lactis* showed a significantly lower risk of GC. A significantly higher Shannon index was observed in the controls than in the patients. The ROC and AUC analysis results suggest that the identified four candidate bacterial species will be clinically useful for the identification of GC in Koreans.

*H. pylori* infection is the strongest single risk factor for GC, specifically in countries where *H. pylori* infection is endemic^15^. Reduced gastric acidity caused by chronic *H. pylori* infection lowers nutrient availability and local innate immunity responses^16^. Corpus-dominant infection leads to gastric mucosal atrophy, with an increase in gastric pH due to the loss of acid-producing parietal cells^17^. Due to the high relative abundance of *H. pylori*, our data represents a unique bacterial profile of the Korean population by virtue of the fact that the Korean people have been exposed to a similar diet for a long period of time^18^. In contrast, a study suggested that *H. pylori* colonization or stomach anatomic sites does not influence the gastric microbiota composition, although it differed between paired nonmalignant and tumor tissues^19^. In our study population, we observed that *H. pylori* had the highest mean relative abundance compared with other bacterial species, specifically in GC patients. The results were similar at the family (*Helicobacteraceae*) and genus (*Helicobacter*) levels. A study that focused on the molecular characterization of the human stomach microbiota in GC patients concluded that *H. pylori* is the dominant member of the nonmalignant gastric tissue microbiota in many GC patients^20^. Interestingly, a study conducted in Colombia revealed that the gastric microbiome composition is considerably different between people but observed a significant correlation with town of origin, although a significant correlation between the *H. pylori* phylogeographic population and microbial composition has not been identified^21^. Thus, the identification of the biological role of *H. pylori* needs to be investigated in clinical practice.

The critical role of *H. pylori* in GC pathogenesis has been well documented. The carcinogenic potential of *H. pylori* can be unraveled due to the effect from two *H. pylori* related virulence factors namely, vacuolating cytotoxin A (VacA) and cytotoxin associated gene A (CagA)^22^. *H. pylori* evasion can be stimulated because of the immunosuppressive activities of VacA which eventually leads to enhance gastric tumor survival^15^. The protein CagA can enter gastric epithelial cells for undergoing phophorylation^15^, leading to structural changes of cells, including cell scattering, elongation^22^, and resistance to apoptosis^23^. Because of changes in physiological and immunological environments in stomach due to the aforementioned *H. pylori* related pathological issues, composition of the gastric microbiota can be altered resulting in a proinflammatory condition^15^. Studies showed a strong association between proinflammatory cytokines polymorphisms and an increased risk of developing *H. pylori*-associated GC^24–26^.

In addition to the carcinogenic role of *H. pylori*, mucosal atrophy plays a critical role in GC pathogenesis^23^. Evidence from our study showed that a higher relative abundance of *H. pylori* increases the risk of GC. Although it has been widely accepted that there is no direct influence of *H. pylori* to the adults’ gastric microbiota composition, few studies have noted that there is an effect owing to the fact that those who carry *H. pylori* are feasible to have higher abundance of *Spirochetes*, *Acidobacteria*, and non-*Helicobacter Proteobacteria* and have comparatively lower abundances of *Actinobacteria*, *Bacteroidetes*, and *Fermicutes* phyla than uninfected adults^27,28^. Our analysis supports the theory that a higher relative abundance of *H. pylori* increases the risk of GC in males although the results are not statistically significant. This finding may be due to our limited sample size, although we had a relatively large overall sample. The Shannon index box plot shows that the alpha diversity was significantly higher in the controls than in the GC patients in our study sample. A similar bacterial diversity has been noted in a study in which there is a higher Shannon index in healthy subjects than in GC patients^29^. Such a change in bacterial composition may be the predominant causes of gastric atrophy resulting in GC even though the number of *H. pylori* bacteria decreases due to atrophy^28^. Additionally, based on our PCoA plot, the bacterial composition at the species level of our study was markedly divergent between the GC patients and control subjects. This finding also resembles the finding observed by Li, T. H. *et al*.^29^, indicating that there is a clear divergence between GC patients and healthy subjects in PCoA analyses.

Regarding *P. acnes*, our study found that individuals with a high relative abundance of *P. acnes* showed an increased risk of GC. A study based on comparative microbial community profiling of human stomach biopsies, found that overabundance of *P. acnes* is a cause of lymphocytic gastritis (LyG)^30^. Our study findings also show the mean relative abundance of *P. acnes* is higher in GC patients than in the controls. The same results were found for both the family (*Propionibacteriaceae*) and genus (*Propionibacterium*) levels. It is notable that *P. acnes*, a classic skin bacterium that causes acne, has been recently identified as a gastric microbiota^31^. Furthermore, LyG caused by *P. acnes* can enhance GC development by producing proinflammatory cytokines such as IL 15^30^. It is important to mention that the gut brain skin hypothesis indicates there is a complex interrelationship between acne and gut dysfunction mediated by the brain which has been recently validated by the microbiome studies^32^. This is supported by the fact that frequent associations of both anxiety and depression and gastrointestinal distress occur with the occurrence of acne. Such condition can be a causative factor for releasing neuropeptides from the enteroendocrine cells due to the production of neurotransmitters, such as serotonin, norepinephrine and acetylcholine by normal flora. These chemicals can trigger both intestinal and systemic inflammation which eventually leads to GC development by increasing the gut permeability that allow for cross talk between the gut and skin^32^.

Recent research in humans found that the localized and systemic diseases, including periodontitis, bacterial vaginosis, rheumatoid arthritis, metabolic disorders, and low grade systemic inflammation can be caused by the overabundance of *Prevotella* species at mucosal sites^33^. *In vitro* study suggested that *Prevotella* has remarkable capability in driving T helper type 17 (Th17) immune responses which evidently propose the association between increased *Prevotella* abundance and augmented Th17^33^. Further, the ability of *Prevotella* in producing redox proteins with an increased resistance to the host has also been proposed^34^. Based on the current study findings, it was observed that the subjects who carried *P. copri* had a significantly higher risk of GC than the subjects who did not carry *P. copri*. Additionally, there was significantly higher mean *P. copri* abundance in the GC patients than in the controls. It has been strongly suggested that *P. copri* induces inflammatory conditions in the human body, leading to the development of several types of diseases, including GC^34^. According to previous study on animal models, it has been shown that the overall bacterial composition can be affected by long term *H. pylori* infection^35^. A relative abundance of the *Prevotella* genera was found in studies conducted with patients without *H. pylori* infection^36^. Thus, *H. pylori*-induced changes in gastric microflora can be attributed to various factors and can lead to gastric atrophy, increased gastric pH, and the colonization of the stomach by transient bacteria^35^. However, the role of *P. copri* in GC development needs to be further investigated.

*L. lactis* was observed as a beneficial bacterium in the current study because the subjects who carried *L. lactis* had a lower GC risk. A study has revealed that there is a strong antiproliferative activity of the cytoplasmic fraction of *L. lactis* upon human colon cancer cells^37^. Furthermore, a study investigating the antiproliferative effects of the cytoplasmic fraction of *L. lactis* on a human stomach cancer cell line revealed that there is an inhibitory effect on cell proliferation with *L. lactis* treatment in a time and dose dependent manner^38^. *L. lactis* caused G0/G1 cell cycle arrest, which was associated with an increase in p53 and p21 expression, a reduction in cyclin D1 expression, and retinoblastoma protein phosphorylation, thereby inducing apoptosis. Additionally, it has been noted that the *L. lactis* bacterium has a probiotic effect in the human gut and can result in beneficial effects for human gastrointestinal health, including the prevention of gastrointestinal cancers^38^. Interestingly, we observed that the mean relative abundance of *L. lactis* was higher in healthy controls than in the GC patients in our study population.

We observed that two linear combinations (*H. pylori* and *P. acnes* dominant) of the four identified candidate bacterial species can be considered predictive of GC, therefore, representing a potential diagnostic marker. Together, our data indicated that *H. pylori* and *P. acnes* were highly abundant in the GC patients and positively identified GC with 79.7% sensitivity. It is evident that the abundance of *H. pylori* becomes lower due to the succession of microbial species as GC locally advances^14^. In contrast, our data indicated that GC patients have significantly high abundances of *H. pylori*. A possible explanation is that we collected the biopsy samples from patients with early gastric cancer in which bacterial succession had not progressed. A study conducted in Taiwan concluded that regardless of the biological roles of *Clostridium*, *Fusobacterium* and *Lactobacillus* in oncogenesis, the overabundance of these microbes serves as a diagnostic tool for GC^14^. Thus, it can be suggested that if there is a bacteria species that can promote the GC occurrence, the eradication of this bacteria is useful for decreasing GC incidence.

The human gut microbiome plays a critical role in gastrointestinal cancers^39^. A report on the next steps in studying the human microbiome and health in prospective studies pointed out that the importance of continuing and expanding of the recent microbiome research^40^. Despite the utilization of optimized techniques for sample collection, processing and storage, supplementary methodological approach is required specifically in the epidemiological context due to the inherent limitations of epidemiological studies. As the potential limitations in case-control studies of the microbiome and cancer, changes of the composition of the microbiome due to dietary factors, selection and recall bias have been emerged. It has been recommended that biorepositories for human samples establish a similar collection method to serve as a basis for nested case-control studies. In addition, conducting short interventions in human and animal studies can identify the important effects of diet on the microbiome, particularly the fecal microbiome^40^. Furthermore, managing optimal storage conditions soon after a sample is collected is necessary to reduce the bias arising from the quick changes in the genetic components of the microbes^41,42^.

Our study has both strengths and limitations. A major strength of our study is that the sample size was relatively large with 268 GC patients and 288 healthy controls, which provides sufficient statistical power to detect the relevant associations between the gastric microbiota and GC risk. Additionally, several possible confounding variables were taken into consideration that are possible risk factors for GC development, including age, smoking, family history of GC, regular exercise, education, occupation, income and total energy intake throughout the analysis. However, our study has potential limitations. In general, the presence of bias associated with a hospital based case-control study, including selection bias and recall bias should be raised. As this is not a prospective study, the associations between microbiome and GC can occur without a causal relation to GC because patients with early GC have altered microbiomes because of atrophy progression. Additionally, we only measured a single sample for our microbial measurements, and it has been shown that microbiome measurements at multiple time points could result in more precise exposure estimates^43^. However, it is important to emphasize that there are ethical issues in repeating biopsies in those with normal gastric histology and healthy subjects.

In conclusion, *H. pylori, P. acnes* and *P. copri* are strong risk factors, whereas *L. lactis* is a protective factor, for GC development in Koreans. The identified four candidate bacterial species will be clinically useful for the identification of GC in Koreans. Further microbiome studies, such as a prospective study that has the capability to infer causality, are warranted to confirm the findings of the current study. Furthermore, the study can be expanded by evaluating the impact of diet on the bacterial composition of the gastric mucosa and by including other races and ethnicities, specifically other East Asian populations, to improve the generalizability of the results. The identification of the gastric bacterial composition can also serve as a readily accessible, noninvasive biomarker for the identification of GC risk^44^. Moreover, conducting GC related pathway and functional studies based on gastric microbiome data is warranted, as most cancer related pathways are yet to be discovered^45,46^.

## Materials and Methods

### Study subjects

Participants were recruited at the National Cancer Center Hospital in Korea between March 2011 and December 2014. Individuals who had been histologically confirmed at the Center for Gastric Cancer as having early GC within the preceding three months were included in the patient group. Early GC was defined as an invasive carcinoma confined to the mucosa and/or submucosa, regardless of lymph node metastasis status. Patients who had been diagnosed with diabetes mellitus or had a history of cancer within the past five years, advanced GC, or severe systemic or mental diseases, as well as women who were pregnant or breastfeeding, were excluded. The control group was selected from individuals undergoing health-screening examinations at the Center for Cancer Prevention and Detection at the same hospital. Individuals in the control group with a history of cancer, diabetes mellitus, gastric ulcers, and *H. pylori* treatment were excluded. The final sample of 556 participants was composed of 268 patients and 288 controls (men, 353; women, 203). All study protocols were conducted according to the Declaration of Helsinki principles. This study was approved by the Institutional Review Board of the National Cancer Center (IRB number: NCCNCS-11-438). Written informed consent was obtained from all participants.

### Data collection

Five gastric mucosa biopsy samples were collected from each study participant following the Sydney system after endoscopy and examination of the stomach. A biopsy sample in the greater curvature, at least 3 cm away from each tumor, was used for the metagenomics analysis. The *H. pylori* infection status was determined by a rapid urease test, a serological test and histological evaluation. Regarding the rapid urease test, one biopsy sample was taken from the greater curvature of the corpus. Four biopsy samples were collected from the lesser curvature of the corpus and antrum for histological evaluation. The *H. pylori* status was determined via Wright-Giemsa staining of the biopsy specimens by a pathologist who specialized in GC. A current infection was defined as at least one positive test result in the rapid urease test or histological evaluation of four biopsy sites^47^.

Participants were asked to complete a self-administered questionnaire. Demographic, lifestyle, physical activity, and medical history data were collected from the participants. Total energy intake was obtained from the semiquantitative food frequency questionnaire (SQFFQ), which has been previously reported as a reliable and valid questionnaire^48^. *H. pylori* infection was assessed by a rapid urease test and histological evaluation.

### DNA extraction

DNA was extracted from the biopsy samples using the MagAttract DNA Blood M48 kit (Qiagen, Hilden, Germany) and BioRobot M48 automatic extraction equipment (Qiagen), according to the manufacturers’ instructions.

### Metagenomic 16S rRNA gene sequencing

Input gDNA (12.5 ng) was amplified with 16S rRNA gene V3-V4 primers, and a subsequent limited cycle amplification step was performed to add multiplexing indices and Illumina sequencing adapters. The final products were normalized and pooled using PicoGreen, and the library sizes were verified using the LabChip GX HT DNA High Sensitivity Kit (PerkinElmer, Massachusetts, USA). Then, we sequenced using the MiSeq™ platform (Illumina, San Diego, USA). Each sequenced sample was prepared according to the Illumina 16S rRNA gene Metagenomic Sequencing Library protocols. DNA quantification and quality were measured by PicoGreen and Nanodrop analyses, respectively. The 16S rRNA genes were amplified using 16S rRNA gene V3-V4 primers for the 288 control samples and the 268 GC patient samples. The primer sequences are as follows: 16S rRNA gene V3-V4 primer.

16S rRNA gene Amplicon PCR Forward Primer.

5′ TCGTCGGCAGCGTCAGATGTGTATAAGAGACAGCCTACGGGNGGCWGCAG.

16S rRNA gene Amplicon PCR Reverse Primer.

5′ GTCTCGTGGGCTCGGAGATGTGTATAAGAGACAGGACTACHVGGGTATCTAATCC.

Preprocessed reads from each sample were used to calculate the number of OTUs. The number of OTUs was determined by clustering the sequences from each sample using a 97% sequence identity cut-off using quantitative insights into microbial ecology (QIIME) software (v.1.8.0). Taxonomic abundance was counted with a National Center for Biotechnology Information (NCBI) database using a confidence threshold of 0.8 derived from the preprocessed reads for each sample. The microbial composition was normalized using the values calculated from the taxonomic abundance count divided by the number of preprocessed reads for each sample to obtain the relative abundance.

### Statistical analysis

To compare the demographic and lifestyle characteristics between the controls and patients, the chi-square test and Student’s *t*-test were performed for categorical variables and continuous variables, respectively. We compared the relative abundance of the bacterial taxa (class, family, genus, and species) between patients and controls. FDR was applied for the multiple comparison correction. The relative abundance of the candidate species was categorized into tertiles based on the relative abundance in the control group. Exceptionally, if more than one third of the subjects has a relative abundance of zero, that bacterial species was categorized into two groups (noncarriers and carriers) based on the median distribution of the controls. Noncarriers were defined as the subjects who had a relative abundance of zero. The group with the lowest relative abundance was used as the reference group. The ORs and 95% CIs were estimated using unconditional logistic regression models. The median values of relative abundance in each tertile category were used as continuous variables to test for trends. The OR estimates were calculated for the crude model (model I) and model II. Model II was adjusted for age, smoking, first-degree family history of GC, regular exercise, education, occupation, monthly income, and total energy intake. An association analysis was performed for the male and female groups. Boxplots were drawn for the Shannon index value comparisons between the patients and controls using the ggplot2 package. A PCoA was performed on a Bray-Curtis dissimilarity based on the OTU relative abundance table for the species level by using the R package’s “vegan”. Sample clustering in beta diversity analysis was tested using ANOSIM. The statistical significance of the observed R was assessed by 10^4^ permutations^9^. To determine whether the candidate bacterial species can be used as diagnostic tool for GC, a ROC curve analysis was performed based on linear discriminant function. Initially, PCA was conducted to obtain two linear combinations of four variables that explain part of the variance of the model. Then, a linear discriminant function was constructed to distinguish between patients with GC and non-GC. For constructing the linear discriminant function, 424 observations were selected as training data set whereas 132 observations were selected as test data set. The training data set was used to calculate the linear discriminant function by using the “MASS” package in R. The model evaluation was performed using the ROC and AUC analysis results. Statistical analyses were conducted by using SAS version 9.4 software (SAS Inc., Cary, NC, USA) and the R platform (version 3.5.1) (The R Foundation for Statistical Computing, Vienna, Austria).

### Ethical statement

This study was approved by the Institutional Review Board of the National Cancer Center and all the methods were performed in accordance with the approved guidelines and regulations (IRB number: NCCNCS-11-438). Written informed consent was obtained from all participants.

## Supplementary information

Supplementary Figure S1

## Data Availability

The sequence data are available https://www.ncbi.nlm.nih.gov/nuccore/KEQH0000000000. All other data used and analyzed during the current study are available from the corresponding author by reasonable request.
