# Author Correction: Association between the relative abundance of gastric microbiota and the risk of gastric cancer: a case-control study

**DOI:** 10.1038/s41598-021-00573-3

**Published:** 2021-10-29

**Authors:** Madhawa Neranjan Gunathilake, Jeonghee Lee, Il Ju Choi, Young-Il Kim, Yongju Ahn, Chanhyeok Park, Jeongseon Kim

**Affiliations:** 1 Department of Cancer Control and Population Health, Graduate School of Cancer Science and Policy, Goyang-si, 10408 Gyeonggi-do South Korea; 2 Department of Cancer Biomedical Science, Graduate School of Cancer Science and Policy, Goyang-si, 10408 Gyeonggi-do South Korea; 3 Center for Gastric Cancer, National Cancer Center Hospital, National Cancer Center, Goyang-si, 10408 Gyeonggi-do South Korea; 4 Microbiome Division, Theragen Etex, 145 Gwanggyo-ro, Gyeongtong-gu, Suwon-si, Gyeonggi-do 16229 South Korea

Correction to: *Scientific Reports* 10.1038/s41598-019-50054-x, published online 19 September 2019

The Data Availability section in the original version of this Article was incomplete.

There, the Data Availability text,

“The data used and analyzed during the current study are available from the corresponding author by reasonable request.”

now reads:

“The sequence data are available https://www.ncbi.nlm.nih.gov/nuccore/KEQH0000000000. All other data used and analyzed during the current study are available from the corresponding author by reasonable request.”

The Article has now been corrected.

