# Supplementary material for: Association between the relative abundance of gastric microbiota and the risk of gastric cancer: a case-control study
Source: Sci Rep. 2019 Sep 19;9:13589. doi: 10.1038/s41598-019-50054-x (PMC6753194; doi:10.1038/s41598-019-50054-x)
Supplement: Supplementary file 1 — Supplementary Figure S1 [file 41598_2019_50054_MOESM1_ESM.pdf]

Supplementary information

Association between the relative abundance of gastric microbiota and the risk of gastric cancer: a case-control study

Madhawa Neranjan Gunathilake[1], Jeonghee Lee[2], Il Ju Choi[3], Young-Il Kim[3], Yongju Ahn[4], Chanhyeok Park[4], Jeongseon Kim[2*]

[1]Department of Cancer Control and Population Health, Graduate School of Cancer Science and Policy, Goyang-si, 10408, Gyeonggi-do, South Korea

[2]Department of Cancer Biomedical Science, Graduate School of Cancer Science and Policy, Goyang-si, 10408, Gyeonggi-do, South Korea

[3]Center for Gastric Cancer, National Cancer Center Hospital, National Cancer Center, Goyang-si, 10408, Gyeonggi-do, South Korea

[4]Microbiome Division, Theragen Etex, 145 Gwanggyo-ro, Gyeongtong-gu, Suwon-si, Gyeonggi-do, 16229, South Korea

***Corresponding Author:**

Jeongseon Kim, PhD**,** Department of Cancer Biomedical Science, Graduate School of Cancer Science and Policy, Goyang-si, 10408, Gyeonggi-do, South Korea. Tel: +82-31-920-2570; Fax: 82-31-920-2579; E-mail: jskim@ncc.re.kr

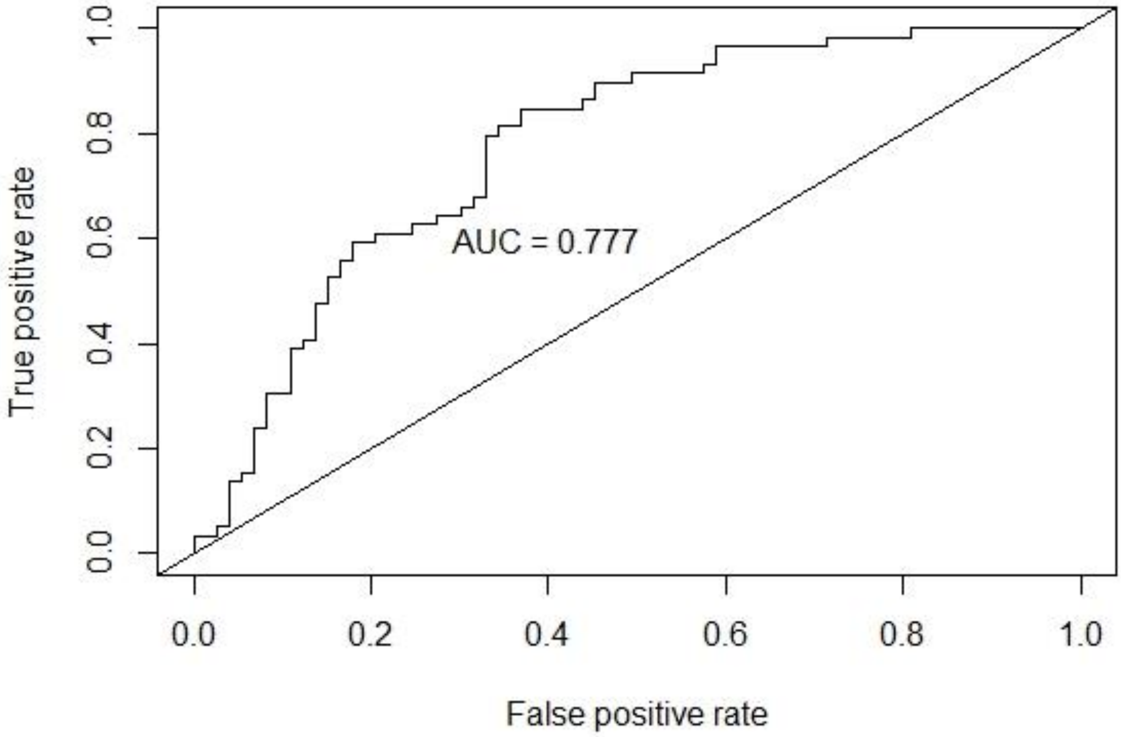

Supplementary Figure S1. Receiver operating characteristic (ROC) curve for GC prediction using two linear combinations of the four bacterial species. An analysis using two linear combinations of four bacterial species showed 79.7% sensitivity and 67.1% specificity. The AUC was 77.7% indicating that there is a 77.7% chance that the model will be able to distinguish between positive and negative classes. The best cutoff point was chosen as 0.492 in order to find a balance between sensitivity and specificity.
